# IL21 is predominantly produced by a CXCL13 associated CD4^+^ T cell subset and shapes the immune microenvironment in colorectal cancer

**DOI:** 10.3389/fimmu.2026.1865519

**Published:** 2026-06-26

**Authors:** Peiyu Lu, Hua Zhou, Hongwei Jiang, You Wu, Hanlin Yang, Yirui Liu, Shaoxian Wu, Min Yang

**Affiliations:** 1 Department of Nephrology, The Third Affiliated Hospital of Soochow University, Changzhou, Jiangsu, China; 2 Department of Tumor Biological Treatment, The Third Affiliated Hospital of Soochow University, Changzhou, Jiangsu, China

**Keywords:** colorectal cancer, CXCL13^+^IL21^+^CD4^+^T cell, IL21, immunotherapy, tumor microenvironment

## Abstract

**Background:**

Interleukin-21 (IL21) is an important immunoregulatory cytokine involved in antitumor immune response; however, its cellular source, regulatory program, and functional significance in colorectal cancer remain incompletely understood.

**Methods:**

We analyzed tumor-infiltrating T cells from the public single-cell RNA sequencing dataset to characterize IL21 expression patterns in colorectal cancer. External validation was performed using a single-cell cohort of colorectal cancer cells treated with anti-PD-1 neoadjuvant therapy. Multiplex immunofluorescence staining was performed on colorectal cancer tissue microarrays to validate the spatial distribution and cellular origin of IL21. Correlation analysis was used to assess the relationship between IL21 and immune-related cell subsets. SCENIC analysis was performed to identify candidate transcription factors associated with CXCL13-associated CD4^+^T cell subsets. Furthermore, tumor tissues from an AOM/DSS-induced mouse colorectal cancer model were cultured *in vitro* (with or without IL21) and batch RNA sequenced to explore IL21-induced transcriptional changes.

**Results:**

Single-cell analysis showed that IL21 expression was restricted to a limited fraction of tumor-infiltrating T cells and was predominantly enriched in CD4^+^T cell subsets, particularly CXCL13^+^CD4^+^T cell populations, whereas IL21R was more broadly expressed. Analysis of the PD-1 anti-neoadjuvant therapy cohort further confirmed that IL21 is also preferentially expressed in specialized CD4^+^ T cell subsets, including CXCL13-associated cell populations. Tissue-level multiplex immunofluorescence confirmed that IL21, IL21^+^CD4^+^T cells, and CXCL13^+^IL21^+^CD4^+^T cells were all significantly increased in tumor tissues compared with adjacent normal tissues. Correlation analysis further suggested that IL21 expression was positively associated with CD4^+^, CXCL13^+^, IL21^+^CD4^+^, and CXCL13^+^IL21^+^CD4^+^T cell populations. SCENIC analysis identified a distinct regulon landscape in the CD4_C09-CXCL13 subset, with STAT1, IRF9, IRF7, TBX21, and IRF4 emerging as candidate key transcription factors. Functionally, exogenous IL21 induced marked transcriptional remodeling in murine colorectal tumor tissues, characterized by activation of immune- and cytokine-related pathways and suppression of multiple Wnt-associated genes.

**Conclusions:**

Our study identifies CXCL13-associated CD4^+^T cells as a major IL21-producing population in colorectal cancer and links this axis to an immune-active tumor microenvironment. Although direct mechanistic validation remains necessary, our findings provide a cellular and transcriptional framework for future investigation of IL21-mediated immune regulation in colorectal cancer.

## Introduction

Colorectal cancer (CRC) remains one of the most common malignancies worldwide and continues to impose a substantial clinical burden despite advances in surgery, chemotherapy, targeted therapy, and molecular stratification (1–3). In recent years, immunotherapy has reshaped the treatment landscape of multiple solid tumors (4); however, in CRC, durable benefit from immune checkpoint inhibitors (ICIs) is largely restricted to the subset of tumors with deficient mismatch repair or microsatellite instability-high status, whereas the majority of patients with microsatellite-stable disease derive limited benefit (5–7). This therapeutic gap has driven growing interest in identifying tumor microenvironmental features that underlie antitumor immunity and may help refine prognostic stratification or uncover new immunoregulatory targets in CRC.

Accumulating evidence indicates that the CRC tumor microenvironment (TME) is highly heterogeneous and that T-cell states, rather than overall lymphocyte abundance alone, may critically determine immune activity and treatment responsiveness. In particular, single-cell technologies have revealed substantial functional diversity among intratumoral T cell populations, including exhausted, cytotoxic, regulatory, tissue-resident, and helper-like subsets (8, 9). Among these, CD4^+^T cells are increasingly recognized as central regulators of antitumor immunity, not only by supporting CD8^+^T cell responses but also by shaping cytokine networks, antigen presentation, and local immune organization within tumors (10–12). These observations suggest that a more precise definition of CD4^+^T cell subpopulations in CRC may provide important biological and translational insights.

Among immune regulatory cytokines, IL21 has emerged as a pleiotropic mediator with important effects on T cells, B cells, NK cells, and other immune populations (13–16). Recent studies and reviews have highlighted the potential of IL21 in tumor immunotherapy, including its ability to enhance type 1 immune responses and its possible value in rational combination strategies with checkpoint blockade (17–19). At the same time, the precise cellular source and spatial context of IL21 expression within CRC remain insufficiently defined. In particular, it is still unclear whether IL21 in CRC reflects a broad inflammatory state or instead marks a specialized immune subset with distinct biological significance. Addressing this question is important because cytokine expression patterns that arise from discrete cellular states may offer more mechanistic information than bulk immune readouts alone.

In parallel, increasing attention has been directed toward CXCL13-associated immune programs in solid tumors. CXCL13 and the CXCL13/CXCR5 axis have been linked to tertiary lymphoid structure biology, immune-active tumor niches, and favorable clinical outcomes in several cancer types (20, 21). In CRC specifically, recent work suggests that elevated CXCL13 expression is associated with a more favorable immune context and improved prognosis, while TLS-related immune organization is increasingly viewed as a biologically and clinically relevant feature of the CRC microenvironment (22–24). These findings raise the possibility that CXCL13-associated helper-like CD4^+^T cell states may represent an important compartment of antitumor immunity in CRC. However, whether IL21 is preferentially linked to such a subset, and how this relationship may be connected to transcriptional regulation and downstream tumor-associated signaling, has not been fully clarified.

Here, we combined single-cell RNA sequencing analysis, multiplex immunofluorescence validation, correlation analysis, SCENIC-based transcriptional inference, and murine ex vivo bulk RNA sequencing to systematically investigate the expression pattern, cellular source, regulatory program, and potential biological role of IL21 in colorectal cancer. Through this integrative approach, we sought to determine whether IL21 is preferentially produced by a specialized CXCL13-associated CD4^+^T cell subset and to clarify the potential significance of this axis in shaping the colorectal cancer immune microenvironment.

## Results

### Single-cell transcriptomic analysis identifies IL21-expressing CD4^+^ T cell subsets in colorectal cancer

**Figure 1:**
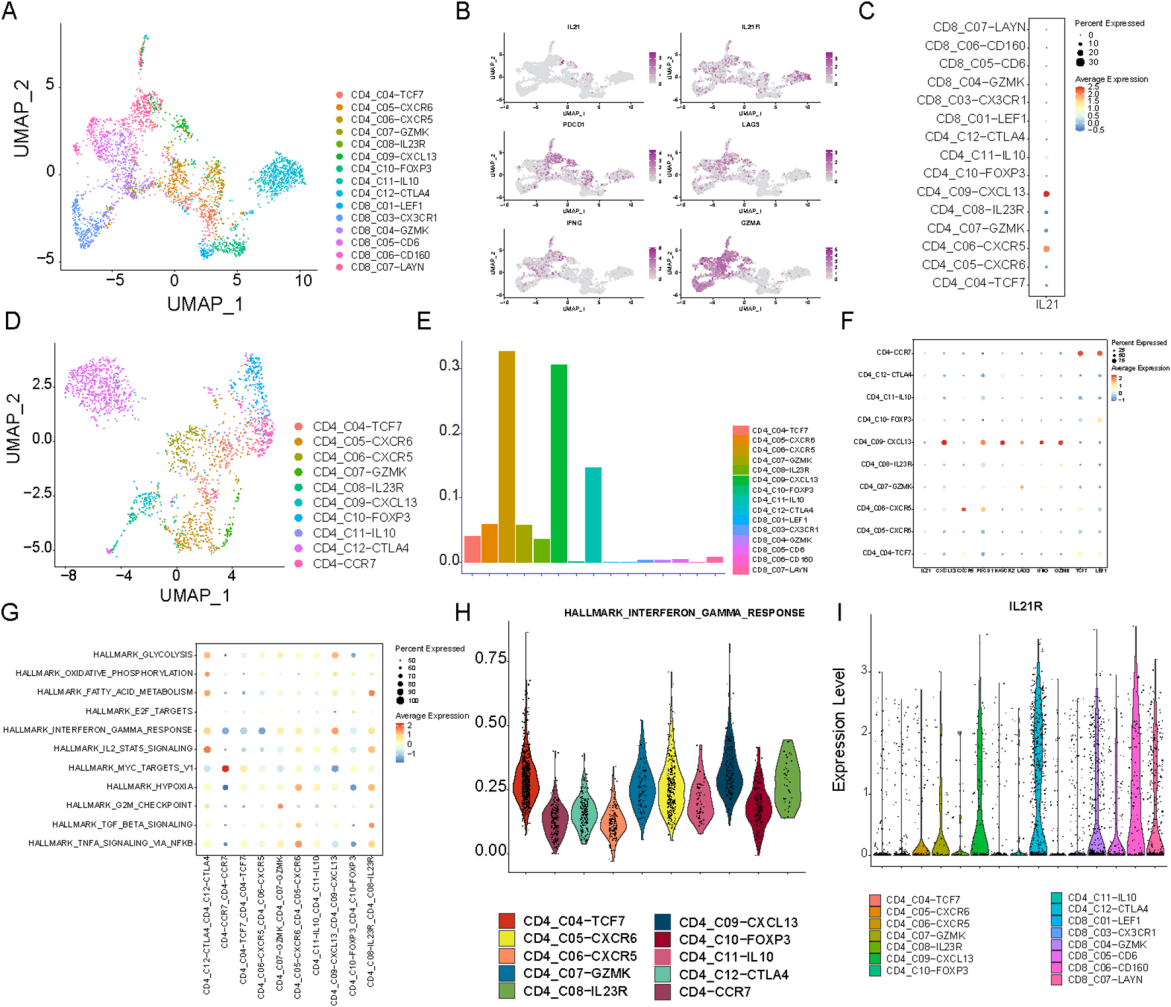
Single-cell transcriptomic analysis reveals IL21-expressing CD4^+^T cell subsets in colorectal cancer. **(A)** UMAP plot showing the clustering of tumor-infiltrating T cells from the GSE108989 dataset. **(B)** Feature plots showing the expression of IL21, IL21R, PDCD1, LAG3, IFNG, and GZMA across T cells. **(C)** Dot plot showing IL21 expression across T-cell subsets. **(D)** UMAP plot of re-clustered CD4^+^T cells. **(E)** Relative proportions of CD4^+^T cell subsets. **(F)** Dot plot showing the expression of representative marker genes in CD4^+^T cell subsets. **(G)** Hallmark pathway activity across CD4^+^T cell subsets. **(H)** Violin plot showing the interferon-γ response score in CD4^+^T cell subsets. **(I)** Violin plot showing IL21R expression across T-cell subsets. Overall, IL21 was mainly enriched in CXCL13^+^/CXCR5^+^CD4^+^T cell subsets, whereas IL21R was more broadly expressed across the T cell compartment.

To investigate the expression pattern of IL21 in tumor-infiltrating T cells from colorectal cancer, we extracted T cells from the GSE108989 single-cell RNA-sequencing dataset and performed unsupervised clustering analysis. UMAP visualization identified multiple transcriptionally distinct T-cell subsets, including diverse CD4 and CD8 populations, highlighting the marked heterogeneity of intratumoral T cells (**Figure 1A**). IL21 expression was confined to a small subset of T cells, whereas IL21R showed a broader distribution across the T-cell compartment (**Figure 1B**). In parallel, activation/exhaustion-associated genes, including PDCD1, LAG3, IFNG, and GZMA, exhibited subset-specific expression patterns, suggesting that IL21 expression is associated with a distinct activated T cell state.

We also found that IL21 was predominantly enriched in CD4^+^T cell subsets, with the strongest expression observed in CD4_C09-CXCL13 and detectable expression in CD4_C06-CXCR5, whereas most CD8^+^T cell clusters showed minimal IL21 expression (**Figure 1C**). These data indicate that IL21 production in colorectal cancer is mainly confined to a specialized CD4^+^T cell population.

To further characterize the cellular source of IL21, we re-clustered CD4^+^T cells and identified several distinct subsets (**Figures 1D, E**). Marker gene analysis showed that the IL21-enriched subsets, particularly CD4_C09-CXCL13 and CD4_C06-CXCR5, co-expressed CXCL13, CXCR5, and PDCD1, together with partial expression of HAVCR2 and LAG3, supporting the CXCL13-associated IL21-producing CD4^+^T cells exhibited a mixed transcriptional profile characterized by helper-like/Tfh-like features, activation-related genes, and exhaustion-associated checkpoint molecules (**Figure 1F**). Functional scoring analysis further revealed substantial heterogeneity in pathway activity across CD4^+^T cell subsets. Hallmark gene set analysis showed differential enrichment of inflammatory and activation-related pathways, and the interferon-γ response signature was relatively elevated in activated CD4^+^T cell subsets (**Figures 1G, H**). In addition, IL21R expression was detected in multiple T-cell subsets (**Figure 1I**), suggesting that IL21 may mediate both autocrine and paracrine signaling within the colorectal cancer immune microenvironment. Collectively, these results indicate that IL21 is mainly expressed by a distinct CXCL13/CXCR5 CD4^+^T cell subsets in colorectal cancer.

### Multiplex immunofluorescence validates the enrichment of IL21-positive cells in colorectal cancer tissues

**Figure 2:**
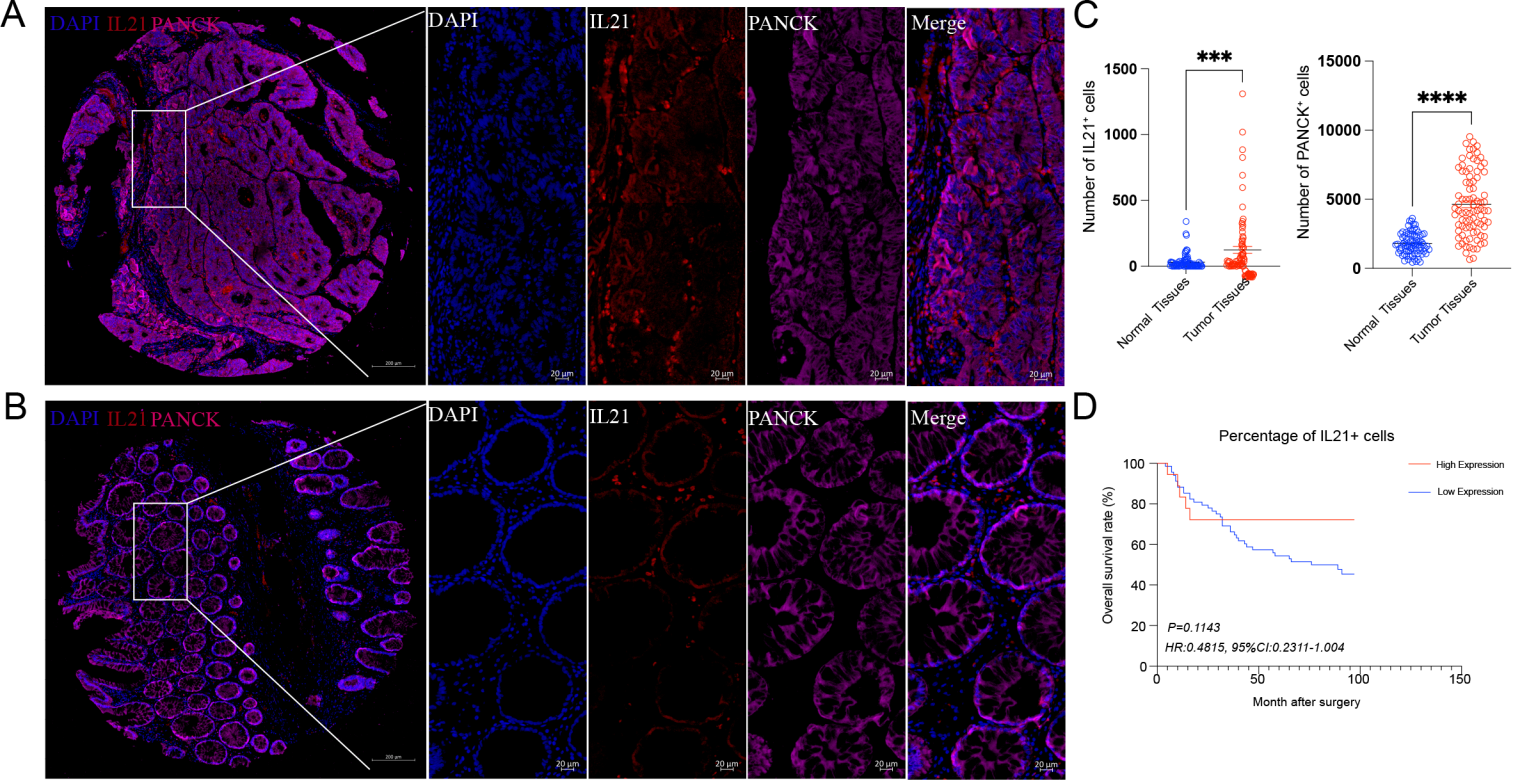
Multiplex immunofluorescence staining validates increased IL21-positive cell infiltration in colorectal cancer tissues. **(A)** Representative images of colorectal cancer tissues stained for DAPI (blue), IL21 (red), and PANCK (magenta), with enlarged views of the boxed regions shown on the right. **(B)** Representative images of adjacent normal tissues stained for DAPI, IL21, and PANCK. **(C)** Quantification of IL21-positive cells and PANCK-positive cells in normal and tumor tissues. **(D)** Kaplan–Meier analysis of overall survival according to the percentage of IL21-positive cells. High IL21 expression was associated with a trend toward improved overall survival. **P < 0.01; ***P < 0.001.

To validate the single-cell findings at the tissue level, we next performed multiplex immunofluorescence staining for IL21, DAPI, and PANCK on a colorectal cancer tissue microarray containing paired tumor and adjacent normal tissues. IL21-positive cells were more abundant in tumor tissues than in adjacent normal tissues (**Figures 2A, B**). Notably, IL21 signals were mainly distributed in the stromal/peri-epithelial regions and showed limited overlap with PANCK-positive epithelial cells, suggesting that IL21 is primarily derived from non-epithelial cells in the tumor microenvironment rather than tumor cells themselves.

Quantitative analysis confirmed that the number of IL21-positive cells was significantly increased in tumor tissues compared with adjacent normal tissues (**Figure 2C**). In parallel, PANCK-positive cells were also markedly higher in tumor tissues, consistent with the expansion of malignant epithelial components. These findings further support the enrichment of IL21-associated immune cells in colorectal cancer tissues.

To assess the clinical significance of IL21 infiltration, patients were stratified according to the percentage of IL21-positive cells and subjected to Kaplan–Meier survival analysis. Patients with high IL21 expression exhibited a trend toward better overall survival than those with low IL21 expression (**Figure 2D**), although the difference did not reach statistical significance (*P = 0.1143; HR = 0.4815, 95% CI: 0.2311–1.004*). Collectively, these results, together with the single-cell data, indicate that IL21-positive immune cells are enriched in colorectal cancer tissues and may be associated with a favorable clinical outcome.

### Single-cell analysis of anti-PD-1 cohort further supports restricted IL21 expression in specialized CD4^+^ T cell subsets

To further validate the robustness of our findings in an independent immunotherapy-treated cohort, we analyzed the single-cell RNA-sequencing dataset GSE236581, which comprises tumor samples from patients receiving anti-PD-1 neoadjuvant therapy. After quality control and unsupervised clustering, all cells were classified into major cellular compartments, including epithelial cells, stromal cells, T cells, B cells, myeloid cells, and ILCs (**Supplementary Figure 1A**). Cell-type annotation was supported by canonical marker genes, including EPCAM for epithelial cells, COL1A1 for stromal cells, CD3D for T cells, MS4A1 for B cells, LYZ for myeloid cells, and NKG7 for ILC-related populations (**Supplementary Figure 1B**).

Analysis of global gene expression patterns showed that IL21 expression was highly restricted and detectable only in a very limited fraction of cells (**Supplementary Figure 1C**), whereas IL21R exhibited a broader distribution across the cellular landscape (**Supplementary Figure 1D**). Re-clustering of the T cell compartment further identified several major T-cell states, including CD8, CD4, cycling T cells, gdT (**Supplementary Figure 1E**), which were validated by the expression of representative markers such as CD8A, CD4, MKI67, and TRDC (**Supplementary Figure 1F**). Within this compartment, IL21 remained confined to a small subset of T cells (**Supplementary Figure 1G**), while IL21R was more widely expressed (**Supplementary Figure 1H**), consistent with the notion that IL21 is produced by a specialized T-cell population but may act on a broader set of cells.

Further subclustering of CD4^+^T cells revealed multiple transcriptionally distinct subsets (**Supplementary Figure 1I**), including CXCL13 associated CD4^+^T cell populations, such as c08_CD4_Tfh_CXCL13_IL6ST and c09_CD4_Th1_CXCL13_HAVCR2. Dot plot analysis showed that IL21 expression was detectable only in a limited fraction of CD4^+^T cells across clinical groups (**Supplementary Figures 1J, K**), rather than being broadly expressed throughout the CD4 compartment. These findings are in line with our main results and further support the conclusion that IL21 is preferentially expressed by a specialized Tfh-like CD4^+^T cell subset, particularly one associated with CXCL13-related features, even in the setting of anti-PD-1 neoadjuvant therapy.

### Multiplex immunofluorescence further identifies IL21^+^CD4^+^ T cells as an enriched IL21-producing population in colorectal cancer

**Figure 3:**
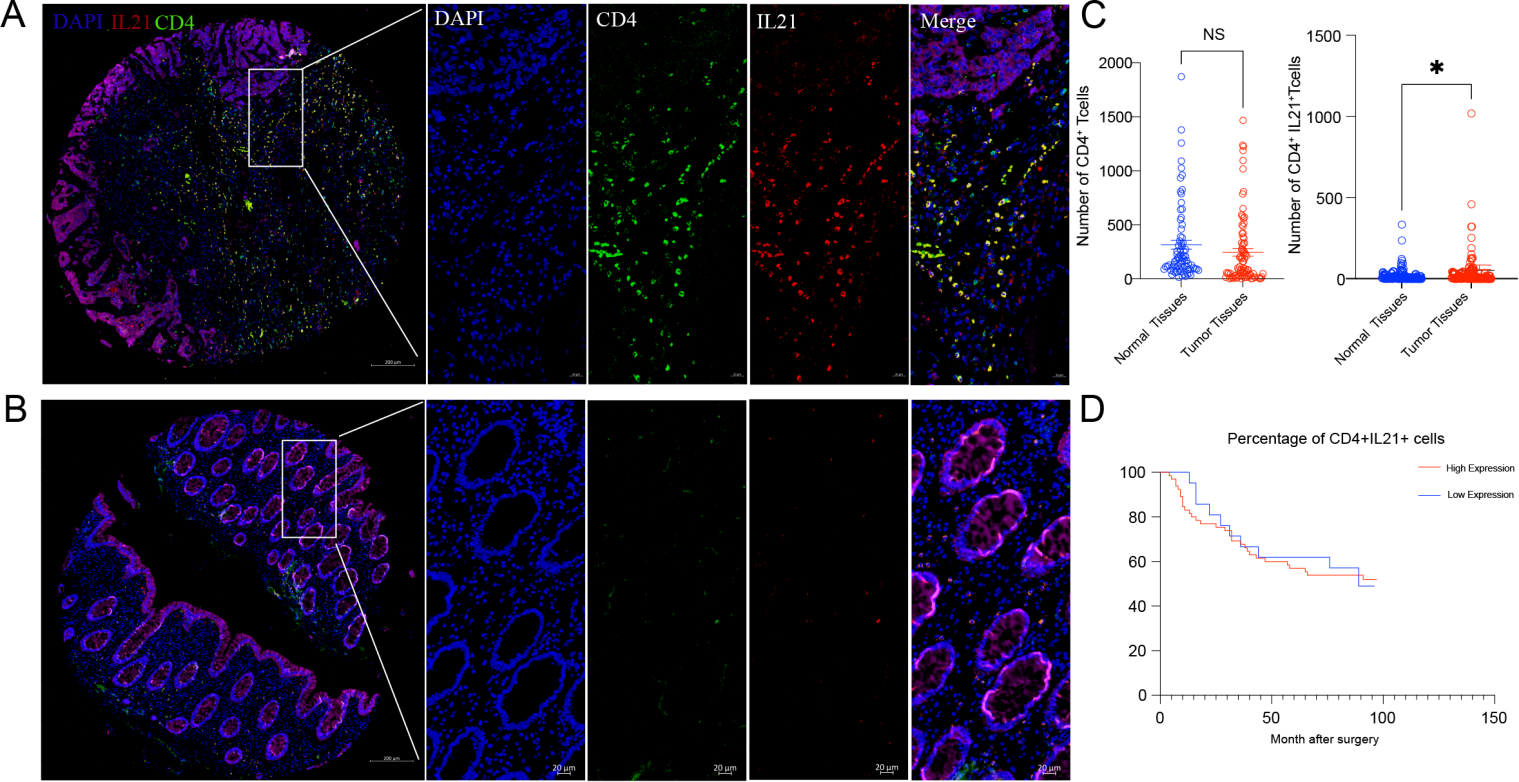
Multiplex immunofluorescence analysis identifies IL21^+^CD4^+^ T cells as an enriched IL21-producing population in colorectal cancer tissues. **(A)** Representative multiplex immunofluorescence images of colorectal cancer tissues stained for DAPI (blue), CD4 (green), and IL21 (red), with enlarged views of the boxed regions shown on the right. **(B)** Representative multiplex immunofluorescence images of adjacent normal tissues stained for DAPI, CD4, and IL21. **(C)** Quantification of CD4^+^T cells and IL21^+^CD4^+^T cells in normal and tumor tissues. Total CD4^+^T cell numbers were not significantly different, whereas IL21^+^CD4^+^T cells were significantly increased in tumor tissues. **(D)** Kaplan–Meier analysis of overall survival according to the percentage of IL21^+^CD4^+^T cells. No significant difference in overall survival was observed between the high- and low-expression groups. *P < 0.05; NS, not significant.

To further identify the cellular source of IL21 in colorectal cancer tissues, we performed multiplex immunofluorescence staining for CD4, IL21, and DAPI on the tissue microarray. IL21^+^CD4^+^T cells were detected in both tumor and adjacent normal tissues, but were more abundant in tumor tissues. Higher-magnification images showed clear co-localization of CD4 and IL21, indicating that a substantial proportion of IL21-expressing cells are CD4^+^T cells (**Figures 3A, B**).

Quantitative analysis showed that the total number of CD4^+^T cells was not significantly different between tumor and adjacent normal tissues, whereas the number of IL21^+^CD4^+^T cells was significantly increased in tumor tissues (**Figure 3C**). These data suggest that the elevated IL21 signal in colorectal cancer tissues is mainly attributable to an enriched IL21-producing CD4^+^T cell population, rather than to a global increase in CD4^+^T cell infiltration. Kaplan–Meier analysis based on the percentage of IL21^+^CD4^+^T cells showed no significant association with overall survival (**Figure 3D**). Taken together, these results further support previous findings from single-cell transcriptome sequencing that IL21 in colorectal cancer is primarily associated with the CD4^+^ T cell population.

### Multiplex immunofluorescence identifies CXCL13^+^IL21^+^CD4^+^T cells as a distinct and enriched T-cell population in colorectal cancer

**Figure 4:**
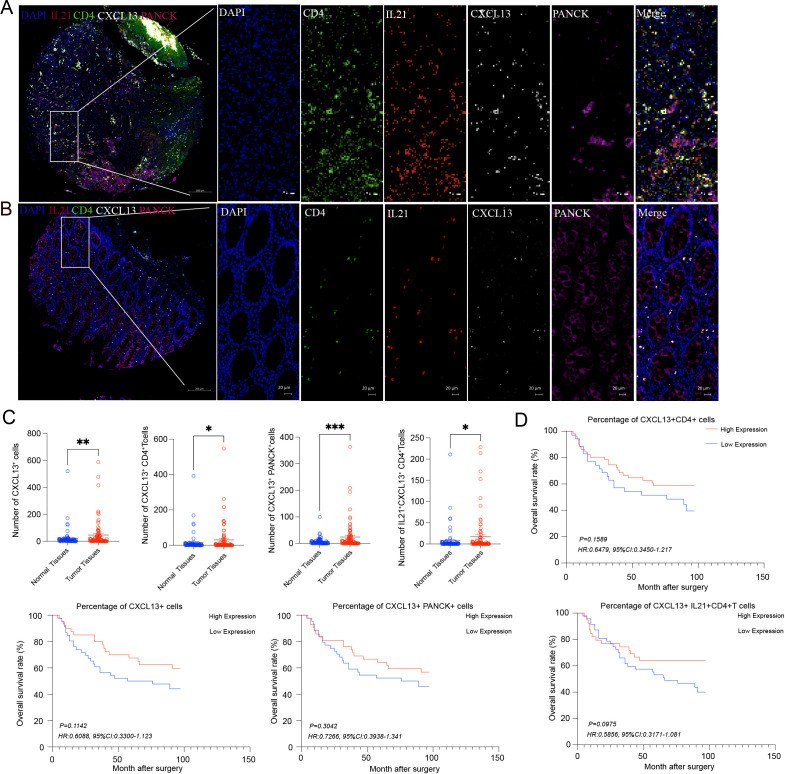
Multiplex immunofluorescence identifies CXCL13^+^IL21^+^CD4^+^T cells as an enriched immune cell population in colorectal cancer tissues. **(A, B)** Representative multiplex immunofluorescence images of colorectal cancer tissues **(A)** and adjacent normal tissues **(B)** stained for DAPI (blue), IL21 (red), CD4 (green), CXCL13 (white), and PANCK (magenta), with enlarged views of the boxed regions shown on the right. **(C)** Quantitative analysis of CXCL13^+^ cells, CXCL13^+^CD4^+^Tcells, CXCL13^+^PANCK^+^ cells, and CXCL13^+^IL21^+^CD4^+^T cells in normal and tumor tissues. **(D)** Kaplan–Meier survival analysis according to the proportions of CXCL13^+^ cells, CXCL13^+^PANCK^+^ cells, and CXCL13^+^CD4^+^ T cells. *P < 0.05; **P < 0.01; ***P < 0.001.

Given that the single-cell data implicated a CXCL13-associated CD4^+^ T cell subset as the major source of IL21, we next sought to further refine the phenotype of IL21-producing CD4^+^T cells in tissue sections by introducing CXCL13 as an additional marker. Multiplex immunofluorescence staining for DAPI, IL21, CD4, CXCL13, and PANCK showed that tumor tissues contained abundant CXCL13-positive cells, among which a subset clearly co-localized with CD4 and IL21, identifying a distinct CXCL13^+^IL21^+^CD4^+^T cell population *in situ* (**Figure 4A**). These triple-positive cells were mainly distributed in stromal regions adjacent to epithelial structures, supporting their immune-cell origin. In adjacent normal tissues, the abundance of CXCL13^+^IL21^+^CD4^+^T cells was markedly lower and the co-localization signals among CXCL13, CD4, and IL21 were comparatively sparse (**Figure 4B**). Quantitative analysis further suggested that the numbers of CXCL13^+^ cells, CXCL13^+^CD4^+^T cells, CXCL13^+^PANCK^+^ cells, and CXCL13^+^IL21^+^CD4^+^T cells were all significantly increased in tumor tissues compared with adjacent normal tissues (**Figure 4C**). Importantly, the enrichment of CXCL13^+^IL21^+^CD4^+^T cells provides direct tissue-level evidence that the IL21-producing CD4^+^T cell population identified by single-cell transcriptomic analysis is expanded in colorectal cancer. To assess the clinical relevance of these CXCL13-related populations, Kaplan–Meier survival analyses were performed after stratifying patients according to the proportions of CXCL13^+^ cells, CXCL13^+^PANCK^+^ cells, and CXCL13^+^CD4^+^T cells. Although higher infiltration of these populations generally tended to correlate with improved overall survival, none of the comparisons reached statistical significance. It is noteworthy that the CXCL13^+^IL21^+^CD4^+^T cells showed a favorable survival trend in the tissue microarray cohort, but given the limited cohort size and the exploratory nature of the analysis, these associations should be interpreted with caution (**Figure 4D**).

Taken together, these results suggest that IL21 in colorectal cancer is mainly produced by a distinct CXCL13 associated CD4^+^T cell population, and that CXCL13^+^IL21^+^CD4^+^T cells represent an enriched immune subset within the colorectal cancer microenvironment. This population likely constitutes a key tissue counterpart of the CXCL13^+^/CXCR5^+^ Tfh-like CD4^+^T cell subsets identified by single cell transcriptomic analysis and may therefore represent an important cellular component of antitumor immune regulation in colorectal cancer.

### Correlation analysis links IL21 expression to CXCL13-associated CD4^+^ immune populations in colorectal cancer

To further characterize the cellular context of IL21 expression in colorectal cancer tissues, we next performed correlation analyses between IL21 and multiple immune- and epithelial-related markers quantified from multiplex immunofluorescence staining. IL21 and immune-cell populations are expressed as the percentage of marker-positive cells among total DAPI^+^ nucleated cells. IL21 expression was positively correlated with the abundance of CD4^+^T cells (*R = 0.3775, P = 0.0003*) and, to a lesser extent, CD8^+^T cells (*R = 0.2450, P = 0.0230*), suggesting that IL21 is associated with CD4^+^T cell infiltration in the colorectal cancer microenvironment. Notably, IL21 showed a stronger positive correlation with CXCL13 (*R = 0.4607, P < 0.0001*) than with total T-cell markers alone. A similarly significant correlation was also observed between IL21 and CXCL13^+^PANCK^+^ cells (*R = 0.5813, P < 0.0001*), indicating that increased IL21 expression is accompanied by elevated CXCL13-related signals within colorectal cancer tissues.

**Figure 5:**
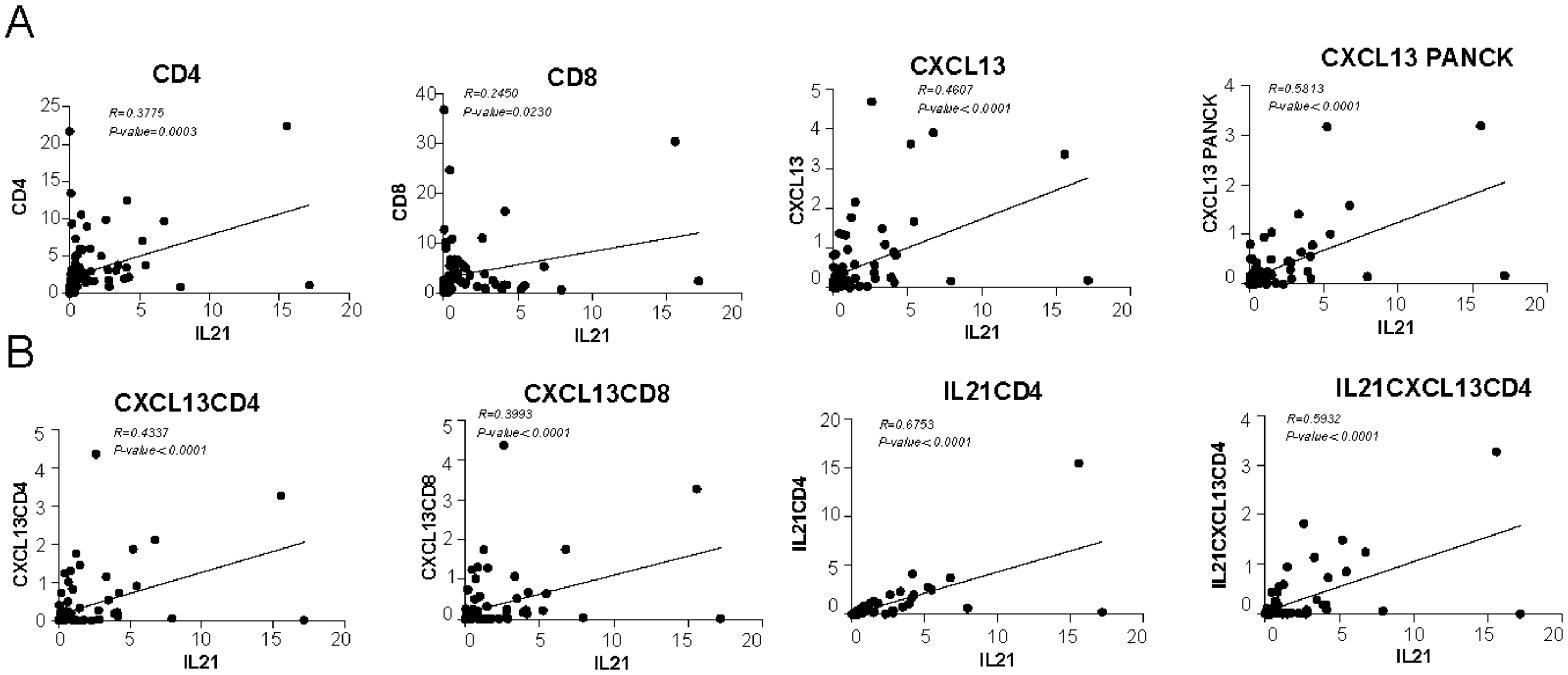
Correlation analysis between IL21 and immune cell markers. **(A, B)** Scatter plots showing the correlations between IL21 expression and the indicated immune cell markers, with correlation coefficients and P values shown in each plot.

When CXCL13-associated lymphocyte subsets were further examined, IL21 was significantly correlated with both CXCL13^+^CD4^+^T cells (*R = 0.4337, P < 0.0001*) and CXCL13^+^CD8^+^T cells (*R = 0.3993, P < 0.0001*). Importantly, the strongest association was observed between IL21 and IL21^+^CD4^+^T cells (*R = 0.6753, P < 0.0001*), which is consistent with our previous findings that CD4^+^T cells represent a major cellular source of IL21 in colorectal cancer tissues. Moreover, IL21 was also strongly correlated with CXCL13^+^IL21^+^CD4^+^T cells (*R = 0.5932, P < 0.0001*), further supporting the existence of a distinct CXCL13-associated IL21-producing CD4^+^ T cell subset in the tumor microenvironment (**Figure 5**). Taken together, these findings reinforce the conclusion that IL21 expression in colorectal cancer is closely linked to CXCL13-related CD4^+^ immune populations, particularly the CXCL13^+^IL21^+^CD4^+^T subset identified above, highlighting this population as a potential key immune component in colorectal cancer.

### Association of IL21 and CXCL13-related immune cell subsets with clinicopathological characteristics and prognosis in colorectal cancer

To further evaluate the clinical significance of IL21 and CXCL13-related immune cell subsets, we analyzed their relationship with clinicopathological features and prognosis in CRC patients (**Supplementary Tables 1–3**). The results showed significant heterogeneity in the correlations between different immune cell subsets and clinical parameters and survival outcomes. In the prognostic analysis, univariate Cox regression results from **Supplementary Table 1** showed that CD4, IL21 expression, CXCL13 expression, IL21^+^CD4^+^, CXCL13^+^CD4^+^, CXCL13^+^CD8^+^, CXCL13^+^PANCK^+^, and CXCL13^+^IL21^+^CD4^+^ were all significantly associated with overall survival. Among them, CXCL13^+^CD4^+^ showed the strongest statistical correlation (*P* = 1.78×10^-5^), followed by CXCL13^+^IL21^+^CD4^+^ (*P* = 3.54×10^-5^), CXCL13^+^CD8^+^ (*P* = 8.77×10^-5^), and CXCL13 expression (*P* = 0.000689). Furthermore, CD4 (*P* = 0.030), IL21 expression (*P* = 0.0212), and IL21^+^CD4^+^ (*P* = 0.00985) were also significantly associated with patient prognosis, while CD8 showed marginal statistical significance (*P* = 0.0574). Further multivariate Cox regression analysis revealed that only CD8 (*P* = 0.000892), CD4^+^ (*P* = 0.001560), and CXCL13^+^CD4^+^T cells (*P* = 0.006696) remained statistically significant, suggesting that CXCL13^+^CD4^+^T cells may have potential prognostic relevance in CRC. However, given the limited TMA cohort size and the exploratory nature of the cutoff-based survival analyses, this result requires validation in larger independent cohorts. Overall, CXCL13^+^CD4^+^T cells were enriched at the tissue level, and exploratory prognostic analyses suggested a possible association between this subset and patient outcomes. These findings indicate that CXCL13^+^CD4^+^T cells may represent a clinically relevant immune population in colorectal cancer, although further validation in larger independent cohorts is required.

Our data also showed that IL21 infiltration levels were significantly correlated with T stage and AJCC stage. In T1–T2 stage patients, the proportion of patients with high IL21 expression was significantly higher than that in T3–T4 stage patients (*P* = 0.037); simultaneously, the distribution of IL21 expression across different AJCC stages also showed significant differences (*P=0.001*). Furthermore, CXCL13 expression was significantly correlated with AJCC stage (*P=0.031*), suggesting that the CXCL13-related immune microenvironment may be involved in colorectal cancer progression. Notably, CXCL13^+^CD4^+^ cell infiltration was significantly correlated with gender, with a higher proportion of high expression in male patients than in female patients (*P* = 0.028). However, CD4^+^ cell infiltration was not significantly correlated with gender, age, tumor size, pathological grade, T stage, or AJCC stage (all *P >*0.05) (**Supplementary Table 2**).

Further analyzed the relationship between CD8^+^, CXCL13^+^CD8^+^, CXCL13^+^PANCK^+^, and CXCL13^+^IL21^+^CD4^+^ cells and clinicopathological features. The results showed that CXCL13^+^CD8^+^ cell infiltration was significantly correlated with gender and pathological grade, with a higher proportion of high expression of CXCL13^+^CD8^+^ cells in male patients than in female patients (*P* = 0.046), and the distribution difference of this cell subset across different pathological grades was also statistically significant (*P* = 0.029). Furthermore, CXCL13^+^PANCK^+^ cell infiltration was significantly correlated with age, with a higher proportion of high expression in patients >58 years of age than in patients ≤58 years of age (*P* = 0.030). In contrast, no significant correlation was observed between CD8^+^ cells and CXCL13^+^IL21^+^CD4^+^ cells and any of the analyzed clinicopathological parameters (all *P>0.05*). These results suggest that the clinicopathological significance of different CXCL13-related cell subsets in CRC is not entirely consistent, and the CXCL13^+^CD8^+^ and CXCL13^+^PANCK^+^ subsets may better reflect the clinical heterogeneity of patients.

### SCENIC analysis identifies key transcription factors associated with the CXCL13^+^ CD4^+^T cell subset in colorectal cancer

To further characterize the transcriptional regulatory program underlying the CXCL13-associated CD4^+^T cell subset, we performed SCENIC analysis across CD4^+^T cell subpopulations identified from the single-cell RNA-sequencing dataset. Regulon activity exhibited substantial heterogeneity among CD4^+^T cell subsets, indicating that distinct CD4^+^T cell populations are governed by different transcription factor networks. Notably, the CD4_C09-CXCL13 subset displayed a unique regulon activity pattern compared with the other CD4^+^T cell clusters, supporting the notion that this population is transcriptionally distinct.

To further define the transcription factors potentially driving the CD4_C09-CXCL13 program, we next focused on differentially enriched regulons. Several transcription factors showed relatively higher activity in the CD4_C09-CXCL13 subset than in most other CD4^+^T cell populations, including STAT1, IRF9, IRF7, TBX21, IRF4, ETS1, CEBPB, and CEBPD. Among these, STAT1, IRF9, and IRF7 are notable components of interferon-related transcriptional programs, suggesting that the CXCL13^+^CD4^+^T cell subset is under strong inflammatory or interferon-responsive regulation. In parallel, elevated TBX21 activity indicates acquisition of an activated effector-like immune state, whereas increased IRF4 is consistent with sustained T-cell activation and differentiation.

**Figure 6:**
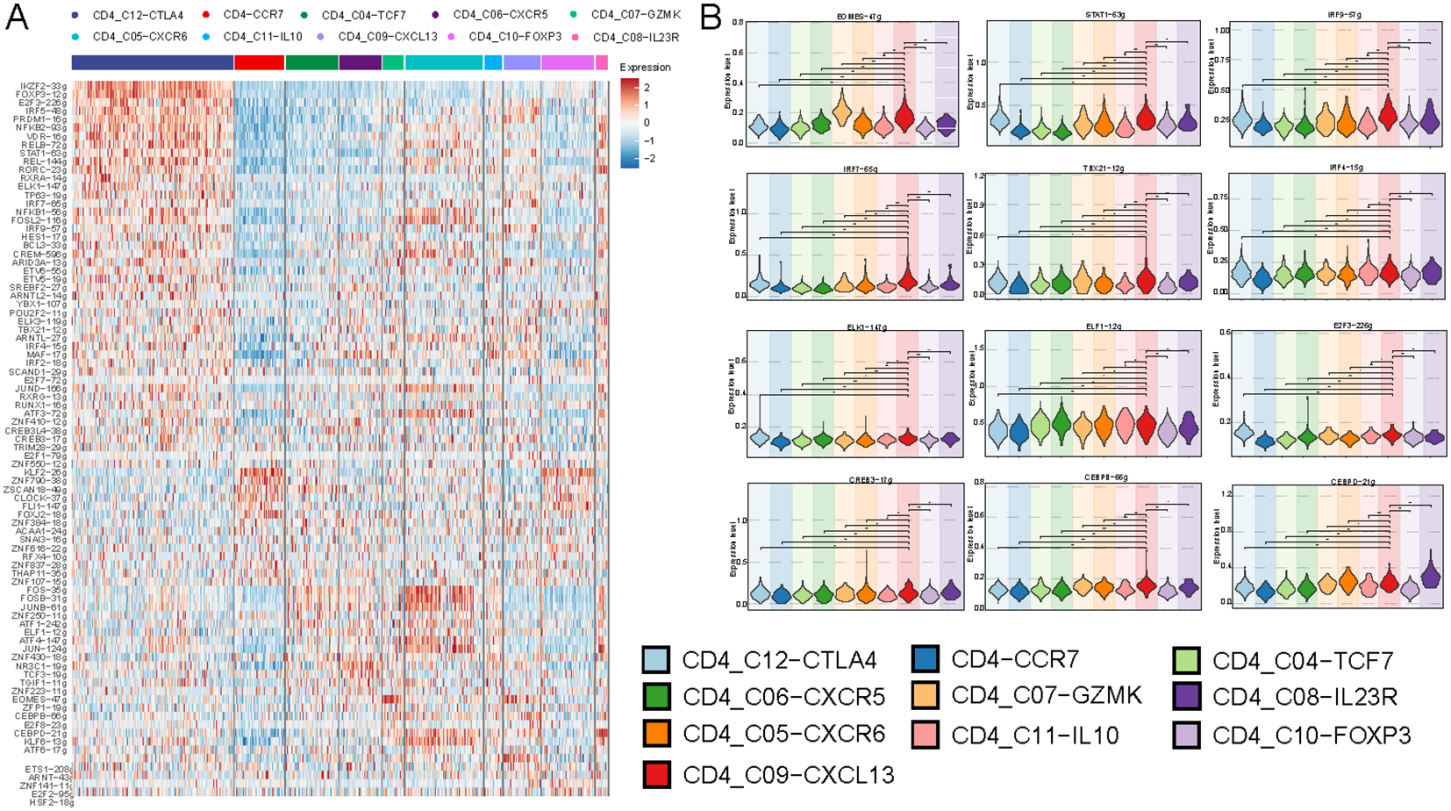
SCENIC analysis reveals distinct transcription factor regulon activity in CD4^+^T cell subsets and identifies candidate regulators of the CXCL13CD4population. **(A)** Heatmap showing differential regulon activity across CD4 T-cell subsets, including CD4_C12-CTLA4, CD4-CCR7, CD4_C04-TCF7, CD4_C05-CXCR6, CD4_C06-CXCR5, CD4_C07-GZMK, CD4_C08-IL23R, CD4_C09-CXCL13, CD4_C10-FOXP3, and CD4_C11-IL10. **(B)** Violin plots showing representative regulon activities across CD4T-cell subsets. The CD4_C09-CXCL13 subset exhibited relatively increased activity of several transcription factors, including STAT1, IRF9, IRF7, TBX21, IRF4, ETS1, CEBPB, and CEBPD, suggesting that interferon-related and activation-associated transcriptional programs are enriched in this population. *P < 0.05; **P < 0.01; ***P < 0.001.

In addition, ETS1, CEBPB, and CEBPD also showed enhanced regulon activity in the CD4_C09-CXCL13 subset, suggesting that this population may integrate inflammatory signaling, activation cues, and lineage-specific transcriptional control to maintain its functional identity. Given that this subset was previously identified as a major IL21-expressing CD4^+^T cell population and showed enrichment of CXCL13/CXCR5/PDCD1-associated features, these transcription factors are likely involved in shaping the Tfh-like and immune-active phenotype of the CXCL13^+^CD4^+^T cell subset compartment (**Figures 6A, B**).

Because SCENIC analysis infers transcriptional regulatory activity from single-cell transcriptomic data, these transcription factors should be interpreted as candidate regulators of the CXCL13-associated CD4^+^T cell program rather than experimentally validated functional drivers. Taken together, the SCENIC analysis shown that the CD4_C09-CXCL13 subset is regulated by a distinct transcription factor network. In particular, STAT1, IRF9, IRF7, TBX21, and IRF4 may represent candidate key transcriptional regulators of this subset, with STAT1/IRF family-associated signaling likely serving as a central regulatory axis for the maintenance of the CXCL13^+^CD4^+^T cell program in colorectal cancer.

### Bulk RNA-seq of IL21-treated murine colorectal tumors reveals extensive transcriptional remodeling and suppression of Wnt-related programs

To further validate the biological effects of IL21 in colorectal cancer, we established an AOM/DSS-induced murine colorectal cancer model. Tumor tissues were harvested, cut into small fragments, and cultured ex vivo in the presence or absence of IL21, followed by bulk RNA sequencing to characterize IL21-induced transcriptional changes.

**Figure 7:**
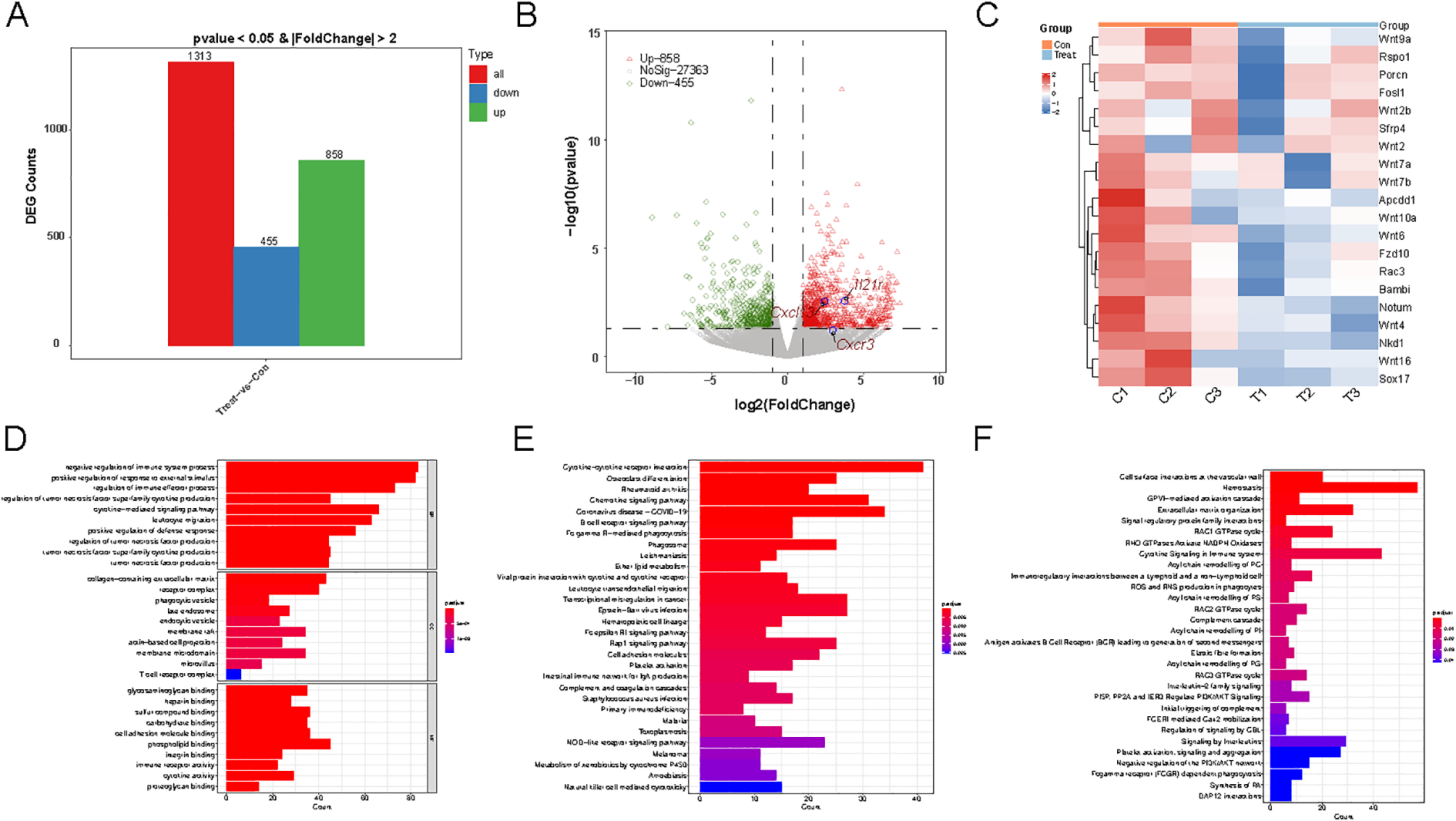
Bulk RNA-seq reveals that IL21 treatment remodels the transcriptional landscape of murine colorectal tumors. **(A)** Differentially expressed gene (DEG) counts between IL21-treated and control tumor tissues from the AOM/DSS-induced murine colorectal cancer model, using the cutoff criteria of *P* < 0.05 and |fold change| > 2. **(B)** Volcano plot showing the distribution of differentially expressed genes between the IL21-treated and control groups. Representative altered genes are indicated. **(C)** Heatmap showing the expression of representative Wnt pathway-related genes in control and IL21-treated tumor tissues. **(D)** GO enrichment analysis of differentially expressed genes after IL21 treatment. **(E)** KEGG pathway enrichment analysis of differentially expressed genes after IL21 treatment. **(F)** Reactome pathway enrichment analysis showing signaling pathways altered by IL21 treatment, including immune regulation-, extracellular matrix-, and interleukin-related pathways.

Differential expression analysis revealed substantial transcriptomic alterations after IL21 treatment. Using the cutoff criteria of *P* < 0.05 and |fold change| > 2, a total of 1,313 differentially expressed genes (DEGs) were identified, including 858 upregulated genes and 455 downregulated genes, indicating that exogenous IL21 induced a marked transcriptional response in murine colorectal tumor tissues (**Figure 7A**). Consistent with this, the volcano plot further illustrated the global distribution of IL21-responsive genes (**Figure 7B**). A large number of genes were significantly upregulated following IL21 treatment, and several representative immune-related genes, including *Il21r*, *Cxcl13*, and *Cxcr3*, were highlighted among the altered transcripts. These findings suggest that IL21 stimulation may reinforce immune-associated signaling and reshape the tumor immune microenvironment at the transcriptional level. Given our previous findings implicating the CXCL13^+^CD4^+^T cell subset as a major IL21-producing population, we next examined the expression of Wnt-related genes after IL21 treatment. Our results indicate that expression of *Wnt9a*, *Rspo1*, *Porcn*, *Wnt2b*, *Wnt2*, *Wnt7a*, *Wnt7b*, *Wnt10a*, *Wnt6*, *Wnt4*, and *Wnt16* was generally reduced, suggesting that IL21 stimulation was associated with reduced expression of several Wnt-associated genes at the tissue level (**Figure 7C**).

GO enrichment analysis showed that IL21-responsive genes were mainly associated with immune system regulation, cytokine-mediated signaling, leukocyte migration, and immune effector processes. Consistently, KEGG analysis revealed enrichment in cytokine–cytokine receptor interaction, chemokine signaling, TNF signaling, and IL-17 signaling pathways, indicating that IL21 strongly remodels immune-related signaling in murine colorectal tumor tissues. Furthermore, Reactome pathway enrichment analysis also identified significant changes in signaling networks related to cell surface interactions at the vascular wall, extracellular matrix organization, signaling by interleukins, cytokine signaling in the immune system, immunoregulatory interactions between a lymphoid and a non-lymphoid cell, and DAP12 signaling (**Figures 7D–F**). Together, these findings indicate that exogenous IL21 stimulation is associated with transcriptional alterations in colorectal tumor tissues, supporting a potential role for IL21 in modulating tumor microenvironment-related programs.

## Discussion

In this study, we integrated single-cell RNA sequencing, multiplex immunofluorescence, correlation analysis, SCENIC inference, and murine ex vivo bulk RNA-seq to define the cellular source and potential function of IL21 in colorectal cancer. Across datasets and experimental platforms, IL21 was not broadly distributed throughout the tumor microenvironment, but was instead enriched in a restricted CD4^+^ T cell subset, particularly within a CXCL13-associated Tfh-like subset. At the tissue level, IL21^+^, CD4^+^IL21^+^, and CXCL13^+^IL21^+^CD4^+^T cells were all increased in tumor tissues relative to adjacent normal tissues, supporting the existence of a specialized IL21-producing immune population in colorectal cancer. CXCL13^+^IL21^+^CD4^+^T cells may have potential prognostic relevance in CRC, although validation in larger independent cohorts is required. These findings argue that IL21 in CRC more likely reflects a discrete immune state than a nonspecific inflammatory signal.

This interpretation is consistent with recent literature positioning IL21 as a biologically important and increasingly translationally relevant cytokine in cancer immunotherapy, including in combination strategies with checkpoint blockade and other immune-modulating approaches (15, 25). Recent studies also support a broader role for CXCL13-associated immune programs in shaping immune-active tumor niches, including tertiary lymphoid structure-related responses and favorable antitumor immunity across solid tumors (26, 27). In colorectal cancer specifically, higher CXCL13 expression has been linked to improved survival and a more favorable immune context, further strengthening the biological plausibility of a CXCL13-centered immune axis in this disease (20). Taken together, our observation that IL21 is concentrated in CXCL13-associated CD4^+^T cell supports the idea that IL21 marks a specialized Tfh-like immune program associated with local immune activation rather than simple T-cell abundance.

External validation in the anti-PD-1 cohort is also noteworthy. Even with immune checkpoint blockade, IL21 remained confined to a small subset of T cells, while IL21R expression was more widespread, suggesting that IL21 may act as a focused immune output, targeting a broader population of reactive cells. This pattern aligns with an emerging view that cytokine-driven helper immune states can persist, albeit in limited quantities, across different therapeutic contexts. More broadly, recent studies have shown that CXCL13-associated immune states remain highly relevant in immunotherapy-treated tumors, although their biological consequences may vary depending on the specific context (28, 29).

A key implication of our study is that the IL21-producing CXCL13-associated CD4^+^ T cell subset appears transcriptionally specialized. SCENIC analysis identified STAT1, IRF9, IRF7, TBX21, and IRF4 as candidate regulators of the CD4_C09-CXCL13 program, suggesting a network enriched for interferon responsiveness and T-cell activation. This is biologically plausible because recent work has shown that CXCL13^+^CD4^+^T cells in tumor-associated niches can display activated, clonally expanded, and microenvironment-shaping features (26, 30). In parallel, the IL21/IL21R axis has been shown to regulate mucosal CD4^+^T cell effector function in the colon, including IFN-γ-associated responses, supporting the relevance of IL21 signaling to intestinal immune regulation more broadly (31). However, the survival and subgroup analyses based on the tissue microarray cohort should be interpreted with caution because of the relatively limited sample size, potential multiple-testing burden, and risk of overfitting. Therefore, these findings are best regarded as exploratory evidence supporting the clinical relevance of IL21-related immune features, and further validation in larger independent cohorts is required. Together, these data support the view that the CXCL13-associated IL21-producing CD4^+^T cell population is not merely a descriptive cluster, but a functionally meaningful immune subset with a distinct regulatory identity.

The transcriptional identity of CXCL13-associated IL21-producing CD4^+^ T cells should be interpreted with caution. Rather than representing a uniformly exhausted or terminally dysfunctional population, this subset displayed mixed transcriptional features, including activation-related, Tfh-like/helper-like, and exhaustion-associated programs. The expression of CXCL13, IL21, ICOS, CD40LG, and related helper-associated genes suggests a Tfh-like or helper-like phenotype, whereas the concomitant expression of immune checkpoint molecules, such as PDCD1, CTLA4, TIGIT, LAG3, and TOX, indicates antigen experience and exhaustion-associated signaling. However, in tumor-infiltrating CD4^+^T cells, checkpoint molecule expression does not necessarily indicate terminal exhaustion, as these markers may also reflect recent activation, chronic antigen stimulation, tissue adaptation, or Tfh-like differentiation.

Our findings are consistent with emerging evidence that CXCL13-expressing T-cell populations in tumors are heterogeneous and may encompass tumor-reactive, helper-like, Tfh-like, progenitor exhausted, and TLS-associated programs (27, 32, 33).

In non-small cell lung cancer, CD4^+^PD-1^+^CXCL13^+^T cells have been described as a helper-like population that interacts with antigen-presenting cells and contributes to shaping the tumor immune microenvironment (34). Across tumor types, CXCL13 expression has also been used to identify both precursor-like and terminally differentiated tumor-reactive T-cell states and has been associated with immune checkpoint blockade response (27). In hepatocellular carcinoma, intratumoral CXCL13^+^CH25H^+^IL21^+^PD-1^+^CD4^+^T cells have been reported to be associated with response-related immune niches involving dendritic cells and CD4^+^T cells after PD-1 blockade (32). Moreover, CXCL13-producing CD4^+^T cells or PD-1^+^CXCR5^−^CD4^+^Th-CXCL13 cells have been associated with TLS organization in ovarian cancer and nasopharyngeal carcinoma (30, 33). Therefore, we interpret the IL21-producing CXCL13-associated CD4^+^T-cell subset identified in colorectal cancer as a transcriptionally distinct, antigen-experienced helper-like state potentially related to Tfh-like and TLS-associated programs, rather than as a definitively exhausted or functionally validated effector population.

Our murine validation experiments further suggest that IL21 may contribute to tumor microenvironment remodeling. Following exogenous IL21 stimulation, murine colorectal tumor tissues exhibited broad transcriptional reprogramming, characterized by enrichment of immune- and cytokine-related pathways together with suppression of multiple Wnt-associated genes. While these ex vivo data do not fully capture the complexity of *in vivo* tumor–immune interactions, they support the interpretation that IL21 may function not only as a marker of immune activation, but also as a mediator capable of reshaping tumor-associated signaling. This interpretation is also aligned with recent reviews proposing that IL21 based strategies may enhance antitumor immunity and may be useful in rational combinatorial immunotherapy design (35, 36).

Several limitations should be acknowledged. First, although our integrated single-cell and tissue-level analyses strongly support CD4^+^T cells, particularly the CXCL13-associated subset, as a major source of IL21 in colorectal cancer, the present study does not establish direct causality between CXCL13-associated IL21-producing CD4^+^ T cells and anti-tumor immunity. Our single-cell and multiplex immunofluorescence analyses suggest the enrichment, spatial localization, and immune-related associations of this population, while the ex vivo IL21 stimulation experiment supports the capacity of IL21 to induce tumor-associated transcriptional remodeling. However, these findings remain based on transcriptomic inference, spatial co-localization, correlative analyses, and ex vivo stimulation, and therefore do not prove that endogenous CXCL13^+^IL21^+^CD4^+^T cells are necessary or sufficient to mediate anti-tumor immunity *in vivo*. Second, although some survival analyses showed favorable trends for IL21-related immune populations, several associations did not reach statistical significance, likely reflecting cohort size limitations and the marked biological heterogeneity of colorectal cancer. Third, the transcription factors identified by SCENIC, including STAT1, IRF9, IRF7, TBX21, and IRF4, should be interpreted as candidate regulators inferred from regulon activity rather than experimentally validated functional drivers. Finally, although the murine ex vivo model identifies IL21-responsive transcriptional changes, it does not fully recapitulate chronic tumor evolution, cell-subset-specific interactions, or systemic immunity *in vivo*. Future studies involving IL21 neutralization, depletion or adoptive transfer of CXCL13-associated CD4^+^ T cells, and perturbation of candidate transcription factors will be needed to define the causal mechanisms underlying this immune axis.

Despite these limitations, our study provides a coherent framework for understanding IL21 biology in colorectal cancer. By integrating human single-cell data, tissue validation, external cohort confirmation, and murine functional analysis, we identify CXCL13^+^IL21^+^CD4^+^T cells as a previously underappreciated immune population in CRC. Overall, our findings support a model in which IL21 is mainly produced by a specialized CXCL13-associated Tfh-like CD4^+^T cell subset and may contribute to shaping an immune-reactive colorectal cancer microenvironment. In this context, the IL21^+^CXCL13 associated CD4^+^T cell axis may represent both a biomarker of an immune-active tumor state and a candidate target for future mechanistic and translational investigation.

## Materials and methods

### Patients and tissue specimens

The human colorectal cancer (CRC) tissue microarray (TMA , catalog HColA180Su18) containing paired tumor tissues and adjacent normal tissues was purchased from Shanghai Outdo Biotech Co., Ltd. (Shanghai, China). Survival rate of 93 cases of colonic adenocarcinoma: 93 cancerous points/87 adjacent cancerous points. (Seventh edition) Clinical stages: 1, 2, 3, and 4. Surgery time: June 2007 – April 2008; follow-up time: July 2015. Follow-up period: 7–8 years. Patients were stratified according to the median proportions or counts of indicated immune cell populations for Kaplan–Meier survival analysis and clinicopathological correlation analysis. The study is reviewed and approved by the Clinical Research Ethics Committee at Outdo Biotech (Shanghai, China, SHYJS-CP-1707002).

### Multiplex immunofluorescence staining

Multiplex immunohistochemistry was performed on the colorectal cancer TMA using the AlphaXTSA^®^ 7-color fluorescence staining kit (Catalog No. AXT37100041, Alpha X Bio, China) on an AlphaPainter^®^ X30 automated staining platform, following the manufacturer’s protocol. In brief, tissue sections were dewaxed (Catalog No. DZ2011, Leagene, China), subjected to alkaline antigen retrieval (Catalog No. ZLI-9079, ZSGBBIO, China), and then sequentially incubated with primary antibodies against CD4 (Catalog No. ZM0418, 1:200 dilution), CD8 (Catalog No. ZA0508, 1:200 dilution), IL21 (Catalog No. NBP2-27336, 1:100 dilution), CXCL13 (Catalog No. ab199043, 1:200 dilution), and PANCK (Catalog No. AXB3012, 1:2000 dilution). Each antibody cycle was completed through primary antibody incubation, secondary antibody binding, and tyramide-based signal amplification, and the slides were counterstained with DAPI at the end of the staining procedure.

### Imaging analysis

After mounting with an antifade reagent, whole-slide fluorescence imaging was acquired using a ZEISS AXIOSCAN 7 system, and image processing and quantitative analysis were carried out with HALO 3.5 software. Human normal tonsil tissue was used as the quality-control sample in each staining batch.

### Image acquisition and quantitative analysis

Multispectral images were acquired using the ZEISS AXIOSCAN 7 scanning system and analyzed with HALO 3.5 software (Indica Labs). Following spectral unmixing, each fluorophore signal was separated into individual channels. HALO was then used to quantify the proportions, densities, and co-localization patterns of the indicated cell populations, including single-positive and multi-positive cells, within tumor and stromal areas. DAPI staining was used to identify nuclei, and cell segmentation was performed to distinguish nuclei, cytoplasm, and membrane compartments. PANCK staining was used to define epithelial/tumor regions, whereas PANCK-negative areas were considered stromal regions. Expression signals for IL21, CD4, CD8, CD21and CXCL13 are similarly combined with DAPI to construct separate binary masks for cells expressing these target biomarkers.

Multiplex immunofluorescence images were analyzed using a standardized image quantification workflow. Images were acquired under identical exposure and scanning settings within each staining panel. Regions of interest were selected from representative tumor areas, while necrotic regions, tissue folds, poorly stained areas, and imaging artifacts were excluded from analysis. Cell segmentation was performed based on DAPI-positive nuclei, followed by marker-based phenotyping using the corresponding fluorescence channels. Marker positivity thresholds were determined according to background fluorescence and negative/control regions within the same staining batch and were then applied consistently across all samples in the same panel. CD4^+^T cells were defined as DAPI^+^ nucleated cells with CD4 positivity, and IL21^+^CD4^+^ or CXCL13^+^CD4^+^ cells were identified based on co-localized marker positivity within the same segmented cell region. Quantitative results were expressed as the density of marker-positive cells and/or the proportion of marker-positive cells within the indicated parent population.

### Survival analysis and multifactorial Cox regression model

Continuous multiplex immunofluorescence-derived variables, including the proportions or densities of IL21-positive cells and indicated immune-cell subsets, were used for survival analyses. Refer to our previously published methods (37, 38), For Kaplan–Meier and Cox regression analyses, optimal cutoff values were determined using the surv_cutpoint() function in the R package survminer, which identifies the cutoff that best separates patients according to overall survival based on maximally selected rank statistics. Patients were then dichotomized into high- and low-expression/infiltration groups according to the corresponding cutoff values. Kaplan–Meier survival curves were then generated to evaluate the prognostic significance of each subset by GraphPad Prism 9 software, and univariate Cox regression analysis was performed to assess their individual associations with survival outcomes. In addition, Multivariate Cox regression was performed using the coxph() function from the R package survival. Considering the limited sample size of the TMA cohort and potential collinearity among immune-cell subsets, multivariate models were constructed in a parsimonious manner by including clinicopathological covariates and selected immune variables rather than all immune parameters simultaneously.

### Public scRNA-seq datasets and data preprocessing

Public single-cell RNA-sequencing (scRNA-seq) datasets were used in this study. The CRC T-cell scRNA-seq dataset GSE108989 was downloaded from the Gene Expression Omnibus (GEO) database and used as the primary discovery cohort for analysis of IL21 expression in tumor-infiltrating T cells. In addition, the scRNA-seq dataset GSE236581, which contains tumor samples from patients treated with anti-PD-1 therapy, was used as an external validation cohort. According to the previously described analyses in the manuscript, T cells or CD4^+^T cells were extracted for downstream analyses.

Data processing was performed using R (version 4.4.1). Batch effects were corrected using harmony when necessary. Cell clustering was performed using a graph-based clustering approach, and dimensionality reduction was visualized by uniform manifold approximation and projection (UMAP).

### SCENIC analysis

To infer transcriptional regulatory networks in CD4^+^T cell subsets, SCENIC analysis was performed using the SCENIC/pySCENIC pipeline. Co-expression modules were first inferred using GRNBoost2 or GENIE3, followed by motif enrichment analysis to define regulons. Regulon activity in each cell was quantified by AUCell. Heatmaps and violin plots were generated to visualize differential regulon activity across CD4^+^T cell subsets, with particular focus on the CD4_C09-CXCL13 cluster. Candidate key transcription factors enriched in this subset, including STAT1, IRF9, IRF7, TBX21, IRF4, ETS1, CEBPB, and CEBPD, were identified based on differential regulon activity.

### Correlation analysis

To determine the relationship between IL21 and immune-related cell populations in CRC tissues, Spearman correlation analysis was performed between IL21 and the abundance of CD4, CD8, CXCL13, CXCL13^+^PANCK^+^, CXCL13^+^CD4^+^, CXCL13^+^CD8^+^, IL21^+^CD4^+^, and IL21^+^CXCL13^+^CD4^+^T cell populations. Correlation coefficients and P values were calculated and visualized by scatter plots using GraphPad Prism 9 software.

### Murine AOM/DSS-induced colorectal cancer model and *ex vivo* IL21 stimulation

**AOM/DSS-induced colorectal cancer model**

Female C57BL/6J mice aged 6–8 weeks were maintained under specific pathogen-free (SPF) conditions and used to establish an azoxymethane/dextran sulfate sodium (AOM/DSS)-induced colorectal cancer model. Azoxymethane (AOM; Sigma-Aldrich, A5486) was dissolved in sterile distilled water at 10 mg/mL as a stock solution, aliquoted, and stored at −20 °C. Before use, the stock was diluted in sterile saline to a working concentration of 1 mg/mL. Dextran sulfate sodium (DSS; MP Biomedicals, 160110) was dissolved in autoclaved water at 2.5% (w/v).Before AOM administration, each mouse was weighed three times, and the mean body weight was used to calculate the injection volume accurately. To induce colorectal tumors, mice received a single intraperitoneal injection of AOM (10 mg/kg). One week later, mice were given 2.5% DSS in drinking water ad libitum for 7 days, with fresh DSS solution replaced every other day, followed by 14 days of recovery with regular drinking water. This treatment regimen was defined as one cycle and was repeated for a total of three cycles. At the experimental endpoint, mice were euthanized and colon tumor tissues were collected for subsequent analyses. Animal experiments were approved Protocol No. (2022) kedi108 at The Third Affiliated Hospital of Soochow University.

### *Ex vivo* IL21 stimulation of tumor tissues

Colon tumors were isolated from mice bearing AOM/DSS-induced colorectal tumors under sterile conditions. Briefly, freshly dissected tumor tissues were transferred to sterile 60-mm culture dishes in a biosafety cabinet and gently washed three times with ice-cold phosphate-buffered saline (PBS) to remove surface debris. Tissues were then supplemented with 3 mL complete DMEM and mechanically minced on ice into approximately 1 mm³ fragments using iris scissors.

The tumor culture medium consisted of DMEM supplemented with 10% fetal bovine serum (FBS), 1% penicillin-streptomycin, 1 mmol/L sodium pyruvate (Sigma-Aldrich, P5280), 1× MEM non-essential amino acids (Sigma-Aldrich, TMS-001-C), and 2 mmol/L L-glutamine (Thermo Fisher, 25030081). The medium was prepared fresh and kept pre-chilled before use.

For three-dimensional ex vivo culture, a Matrigel/collagen I-based matrix was used. Briefly, tumor culture medium containing 1.1% (w/v) sodium bicarbonate (Sigma-Aldrich, S5761) was kept cold, and ice-cold collagen I (1 mg/mL; Corning, 354265) and Matrigel (4 mg/mL; Corning, 356231) were slowly added to prepare the matrix mixture. The matrix was maintained on ice throughout the procedure to prevent premature solidification. Flat-bottom 96-well plates were pre-chilled for 15 min, and all plating procedures were performed on ice. A 40 μL aliquot of matrix was first added to each well as the bottom layer and allowed to solidify at 37 °C for 30 min. After gelation, one freshly minced tumor fragment was placed into each well and overlaid with an additional 40 μL of matrix, followed by incubation at 37 °C for another 30 min to allow complete solidification. Once the bilayer matrix had fully solidified, 120 μL tumor culture medium without sodium bicarbonate was added to each well for ex vivo culture.

Recombinant murine IL21 was added to the culture system at the time of plating. According to the experimental design, IL21 was added to the tumor culture medium at a final concentration of 100 ng/mL. Control wells received an equal volume of vehicle. Fresh treatment medium was prepared at each medium change. Cultures were maintained for 1 week, with medium replacement approximately every other day. At the end of the culture period, tissue samples were harvested for bulk RNA sequencing.

### Bulk RNA-seq and differential expression analysis

Total RNA was extracted from tissue samples. The concentration and purity of the extracted RNA were detected using Nanodrop 2000, RNA integrity was detected by agarose gel electrophoresis, and RIN value was determined using Agilent 2100. For a single library preparation, the total RNA amount should be ≥1 μg, the concentration ≥35 ng/μL, OD260/280 ≥1.8, and OD260/230 ≥1.0, and sequencing libraries were prepared using Illumina TruseqTM RNA sample prep Kit library preparation kit. Paired-end sequencing was performed on the Illumina Novaseq 6000 platform.

The raw data obtained from sequencing contains a small number of reads with sequencing adapters or low sequencing quality. In order to ensure the quality and reliability of data analysis, the raw data is filtered using the fastp software, and gene-level counts were generated. Differentially expressed genes (DEGs) between IL21 treated and control groups were identified using DESeq2, with thresholds of *P* < 0.05 and |fold change| > 2. Volcano plots and bar plots were generated to visualize DEGs.

### GO, KEGG, and pathway enrichment analyses

Gene Ontology (GO), Kyoto Encyclopedia of Genes and Genomes (KEGG), and additional pathway enrichment analyses were performed on DEGs identified from murine bulk RNA-seq data using clusterProfiler. Enriched pathways related to immune system regulation, cytokine-mediated signaling, leukocyte migration, chemokine signaling, TNF signaling, IL-17 signaling, extracellular matrix organization, interleukin signaling, negative regulation of the PI3K/AKT network, and Wnt-associated pathways were visualized using bar plots or heatmaps.

### Statistical analysis

All statistical analyses were performed using R software (version 4.3.1), GraphPad Prism 9, and (SPSS29.0.1.0). Kaplan–Meier survival curves and univariate Cox regression were analyzed with GraphPad Prism 9. Multivariate Cox regression, together with bulk RNA-seq, scRNA-seq, and spatial transcriptome data analyses, was carried out using R version 4.3.1 and RStudio. The analysis of clinicopathological features was performed using SPSS software with a chi-square test. Correlation analyses were performed using Spearman or Pearson correlation tests. A two-sided P < 0.05 was considered statistically significant.

## Data Availability

The public single-cell RNA-sequencing datasets analyzed in this study are available in GEO under accession numbers GSE108989 and GSE236581. The bulk RNA-sequencing data generated in this study are currently being deposited in GEO, and we will provide the accession number as soon as it is assigned.

## Supplementary material

Supplementary Figure 1 Single-cell analysis of anti-PD-1 neoadjuvant therapy cohort reveals restricted IL21 expression and identifies CXCL13-associated CD4^+^ T cell subsets. **(A)** UMAP visualization of major cell populations identified from the GSE236581 single-cell RNA-sequencing dataset, including epithelium, stromal cells, T cells, B cells, myeloid cells, and ILCs. **(B)** Feature plots showing canonical marker genes used for cell-type annotation, including EPCAM, COL1A1, CD3D, MS4A1, LYZ, and NKG7. **(C)** Feature plot showing IL21 expression across all cells. **(D)** Feature plot showing IL21R expression across all cells. **(E)** UMAP visualization of re-clustered T-cell populations, including CD8, CD4, cycling T cells, gdT. **(F)** Feature plots showing representative marker genes for T cell subpopulation annotation, including CD8A, CD4, MKI67, and TRDC. **(G)** Feature plot showing IL21 expression in the T-cell compartment. **(H)** Feature plot showing IL21R expression in the T-cell compartment. **(I)** UMAP visualization of re-clustered CD4T-cell subsets. **(J, K)** Dot plots showing IL21 expression across clinical groups/subsets within the CD4^+^T cell compartment. These results indicate that IL21 is expressed in only a limited fraction of CD4^+^T cells, whereas IL21R is more broadly distributed.
