# Supplementary figures and images for: IL21 is predominantly produced by a CXCL13 associated CD4+ T cell subset and shapes the immune microenvironment in colorectal cancer
Source: Front Immunol. 2026 Jun 26;17:1865519. doi: 10.3389/fimmu.2026.1865519 (PMC13350345; doi:10.3389/fimmu.2026.1865519)

A

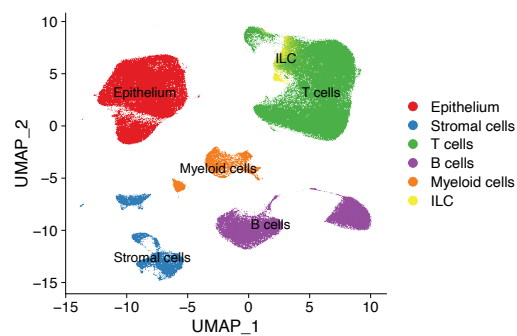

B

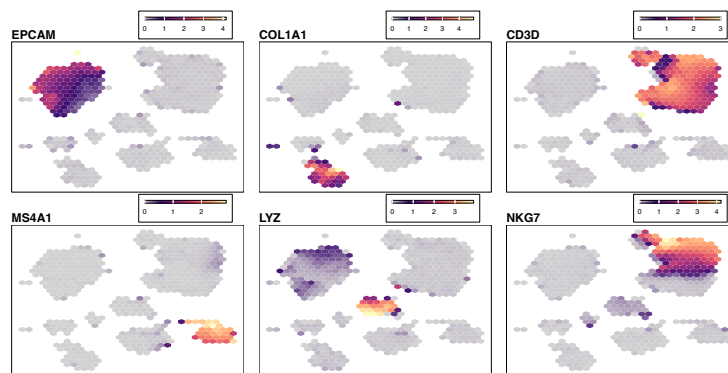

C

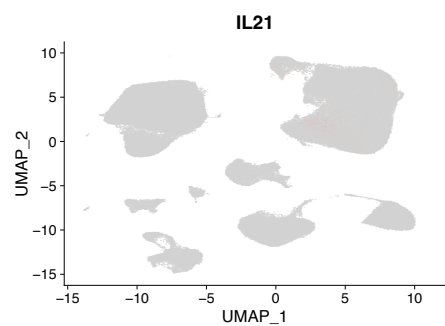

D

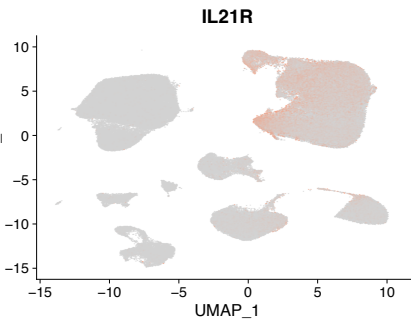

E

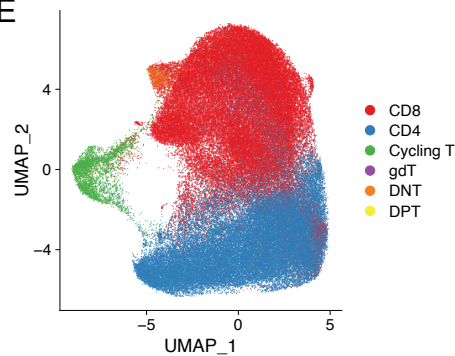

F

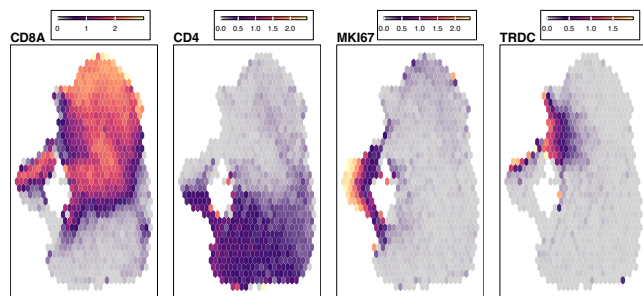

G

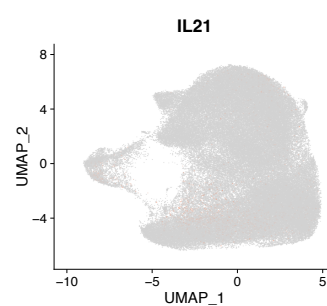

H

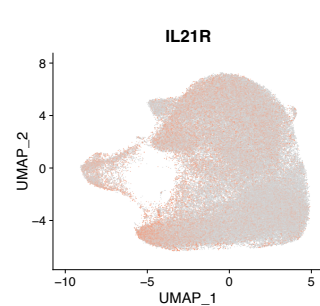

I

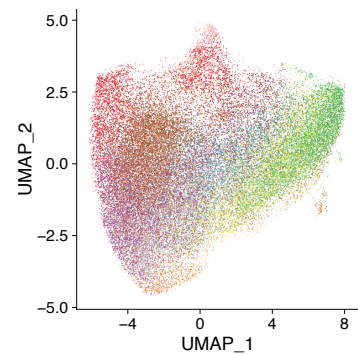

J

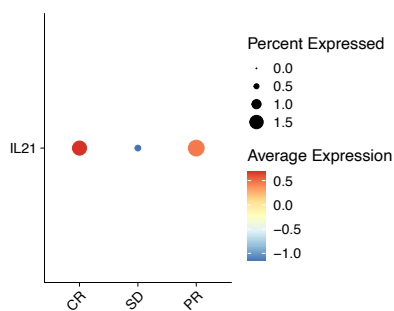

K

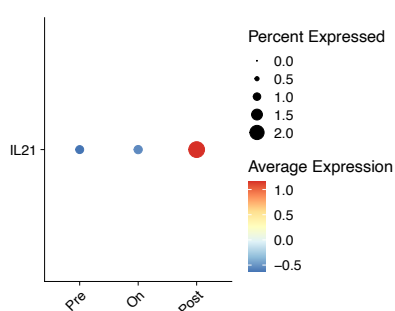

Supplement: Supplementary Figure 1 — Single-cell analysis of anti-PD-1 neoadjuvant therapy cohort reveals restricted IL21 expression and identifies CXCL13-associated CD4+ T cell subsets. (A) UMAP visualization of major cell populations identified from the GSE236581 single-cell RNA-sequencing dataset, including epithelium, stromal cells, T cells, B cells, myeloid cells, and ILCs. (B) Feature plots showing canonical marker genes used for cell-type annotation, including EPCAM, COL1A1, CD3D, MS4A1, LYZ, and NKG7. (C) Feature plot showing IL21 expression across all cells. (D) Feature plot showing IL21R expression across all cells. (E) UMAP visualization of re-clustered T-cell populations, including CD8, CD4, cycling T cells, gdT. (F) Feature plots showing representative marker genes for T cell subpopulation annotation, including CD8A, CD4, MKI67, and TRDC. (G) Feature plot showing IL21 expression in the T-cell compartment. (H) Feature plot showing IL21R expression in the T-cell compartment. (I) UMAP visualization of re-clustered CD4T-cell subsets. (J, K) Dot plots showing IL21 expression across clinical groups/subsets within the CD4+T cell compartment. These results indicate that IL21 is expressed in only a limited fraction of CD4+T cells, whereas IL21R is more broadly distributed. [file DataSheet1.pdf]
