# Supplementary material for: IL21 is predominantly produced by a CXCL13 associated CD4+ T cell subset and shapes the immune microenvironment in colorectal cancer
Source: Front Immunol. 2026 Jun 26;17:1865519. doi: 10.3389/fimmu.2026.1865519 (PMC13350345; doi:10.3389/fimmu.2026.1865519)
Supplement: Supplementary file 3 [file Table1.docx]

| Variable |  | Univariate Cox | |  |  | Multivariate Cox | |  |
| --- | --- | --- | --- | --- | --- | --- | --- | --- |
|  |  | *HR (95% CI)* | *P* |  | *HR (95% CI)* |  | *P* |  |
| Age |  | 1.407(0.356-1.419) | 0.333 |  | 1.42244 (0.177363-2.7865) |  | 0.616024 |  |
| Gender |  | 1.615(0.342-1.120) | 0.113 |  | 2.26620(0.147019-1.3244) |  | 0.144593 |  |
| Tumor size |  | 1.539(0.3587-1.177) | 0.155 |  | 1.48821(0.215746-2.0928) |  | 0.492783 |  |
| Pathological stage (III +IV/I+II) |  | 1.640(0.237-1.568) | 0.304 |  | 1.09658 (0.258124-3.2218) |  | 0.886157 |  |
| T stage (III +IV/I+II) |  | 0.597(0.402-6.971) | 0.479 |  | 1.77709(0.057787-5.4796) |  | 0.620506 |  |
| CD8 |  | 1.777(0.311-1.018) | 0.0574 |  | 10.35458(0.024324- 0.3834) |  | 0.000892 |  |
| CD4 |  | 1.999(0.268-0.934) | 0.030 |  | 0.05713(2.970740-103.1257) |  | 0.001560 |  |
| IL21 expression (high/low) |  | 2.16(0.2405-0.8911) | 0.0212 |  | 0.81254(0.105514-14.3550) |  | 0.868447 |  |
| CXCL13 expression (high/low) |  | 2.866(0.19-0.6409) | 0.000689 |  | 0.14119(0.680211-73.7438) |  | 0.101504 |  |
| IL21CD4 |  | 2.493(0.2005-0.8027) | 0.00985 |  | 1.45123(0.155555-3.0524) |  | 0.623840 |  |
| CXCL13CD4 |  | 3.884(0.1385-0.4785) | 1.78e-05 |  | 41.89632(0.001604-0.3551) |  | 0.006696 |  |
| CXCL13CD4 |  | 3.884(0.1385-0.4785) | 1.78e-05 |  | 41.89632(0.001604-0.3551) |  | 0.006696 |  |
| CXCL13CD8 |  | 3.561(0.1489-0.5297) | 8.77e-05 |  | 0.61353(0.213094-12.4670) |  | 0.637911 |  |
| CXCL13 PANCK |  | 3.195(0.1684-0.5819) | 0.000241 |  | 3.62629(0.035756-2.1268) |  | 0.216473 |  |
| CXCL13IL21CD4 |  | 3.749(0.1426-0.499) | 3.54e-05 |  | 0.26661(0.136303-103.2134) |  | 0.434428 |  |
|  |  |  |  |  |  |  |  |  |

Bold signifies P < 0.05.

**Supplementary Table 1**  Univariate and multivariate Cox regression models
