# Supplementary material for: IL21 is predominantly produced by a CXCL13 associated CD4+ T cell subset and shapes the immune microenvironment in colorectal cancer
Source: Front Immunol. 2026 Jun 26;17:1865519. doi: 10.3389/fimmu.2026.1865519 (PMC13350345; doi:10.3389/fimmu.2026.1865519)
Supplement: Supplementary file 4 [file Table2.docx]

| Variable | Cases |  | IL-21 infiltration degree | | *χ*^2^ | *P* |  | CD4^+^ infiltration degree | | *χ*^2^ | *P* |  | CXCL13 infiltration degree | | *χ*^2^ | *P* |  | CXCL13^+^CD4^+^ infiltration degree | | *χ*^2^ | *P* |
| --- | --- | --- | --- | --- | --- | --- | --- | --- | --- | --- | --- | --- | --- | --- | --- | --- | --- | --- | --- | --- | --- |
|  |  |  | low expression | high expression |  |  |  | low expression | high expression |  |  |  | low expression | high expression |  |  |  | low expression | high expression |  |  |
| Gender |  |  |  |  |  |  |  |  |  |  |  |  |  |  |  |  |  |  |  |  |  |
| Male | 43 |  | 32 | 11 | 0.632 | 0.426 |  | 15 | 28 | 0.438 | 0.508 |  | 18 | 25 | 3.786 | 0.052 |  | 12 | 31 | 4.818 | **0.028** |
| Female | 43 |  | 36 | 7 |  |  |  | 19 | 24 |  |  |  | 28 | 15 | 4.674 | 0.031 |  | 23 | 20 |  |  |
| Age |  |  |  |  |  |  |  |  |  |  |  |  |  |  |  |  |  |  |  |  |  |
| >58 | 71 |  | 54 | 17 | 2.234 | 0.135 |  | 28 | 43 | 0.000 | 1.000 |  | 35 | 36 | 1.991 | 0.158 |  | 28 | 43 | 0.052 | 0.819 |
| ≤58 | 15 |  | 14 | 1 |  |  |  | 6 | 9 | 0.002 | 0.968 |  | 11 | 4 |  |  |  | 7 | 8 |  |  |
| Tumor size |  |  |  |  |  |  |  |  |  |  |  |  |  |  |  |  |  |  |  |  |  |
| >4.8 | 52 |  | 41 | 11 | 0.000 | 1.000 |  | 19 | 33 | 0.228 | 0.633 |  | 25 | 27 | 1.392 | 0.238 |  | 18 | 34 | 1.429 | 0.232 |
| ≤4.8 | 34 |  | 27 | 7 |  |  |  | 15 | 19 |  |  |  | 21 | 13 |  |  |  | 17 | 17 |  |  |
| Pathological stage |  |  |  |  |  |  |  |  |  |  |  |  |  |  |  |  |  |  |  |  |  |
| I-II | 72 |  | 55 | 17 | 1.921 | 0.166 |  | 27 | 45 | 0.332 | 0.564 |  | 37 | 35 | 0.351 | 0.554 |  | 26 | 46 | 2.776 | 0.096 |
| III-IV | 14 |  | 13 | 1 |  |  |  | 7 | 7 |  |  |  | 9 | 5 |  |  |  | 9 | 5 |  |  |
| T stage |  |  |  |  |  |  |  |  |  |  |  |  |  |  |  |  |  |  |  |  |  |
| T1-T2 | 5 |  | 3 | 2 | 4.338 | **0.037** |  | 0 | 5 | 0.004 | 0.947 |  | 3 | 2 | 0.294 | 0.587 |  | 1 | 4 | 0.885 | 0.347 |
| T3-T4 | 80 |  | 64 | 16 |  |  |  | 34 | 46 |  |  |  | 42 | 38 |  |  |  | 33 | 47 |  |  |
| AJCC stage |  |  |  |  |  |  |  |  |  |  |  |  |  |  |  |  |  |  |  |  |  |
| I-II | 53 |  | 42 | 11 | 12.239 | **0.001** |  | 20 | 33 | 0.042 | 0.837 |  | 23 | 30 | 4.647 | **0.031** |  | 19 | 34 | 0.873 | 0.350 |
| III-IV | 33 |  | 26 | 7 |  |  |  | 14 | 19 |  |  |  | 23 | 10 |  |  |  | 16 | 17 |  |  |

Bold signifies P < 0.05.

**Supplementary Table 2** Correlations of IL21, CD4^+^, CXCL13 and CXCL13^+^CD4^+^ expression with clinicopathological parameters in colon cancer patients
