# Supplementary material for: IL21 is predominantly produced by a CXCL13 associated CD4+ T cell subset and shapes the immune microenvironment in colorectal cancer
Source: Front Immunol. 2026 Jun 26;17:1865519. doi: 10.3389/fimmu.2026.1865519 (PMC13350345; doi:10.3389/fimmu.2026.1865519)
Supplement: Supplementary file 5 [file Table3.docx]

| Variable | Cases |  | CD8^+^  infiltration degree | | *χ*^2^ | *P* |  | CXCL13^+^CD8^+^  infiltration degree | | *χ*^2^ | *P* |  | CXCL13^+^PACNK^+^  infiltration degree | | *χ*^2^ | *P* |  | CXCL13^+^IL21^+^CD4^+^  infiltration degree | | *χ*^2^ | *P* |
| --- | --- | --- | --- | --- | --- | --- | --- | --- | --- | --- | --- | --- | --- | --- | --- | --- | --- | --- | --- | --- | --- |
|  |  |  | low expression | high expression |  |  |  | low expression | high expression |  |  |  | low expression | high expression |  |  |  | low expression | high expression |  |  |
| Gender |  |  |  |  |  |  |  |  |  |  |  |  |  |  |  |  |  |  |  |  |  |
| Male | 43 |  | 33 | 10 | 0.061 | 0.805 |  | 12 | 31 | 3.983 | **0.046** |  | 18 | 25 | 2.280 | 0.131 |  | 19 | 24 | 3.003 | 0.083 |
| Female | 43 |  | 31 | 12 |  |  |  | 21 | 22 |  |  |  | 26 | 17 |  |  |  | 28 | 15 |  |  |
| Age |  |  |  |  |  |  |  |  |  |  |  |  |  |  |  |  |  |  |  |  |  |
| >58 | 71 |  | 54 | 17 | 0.574 | 0.449 |  | 26 | 45 | 0.189 | 0.664 |  | 32 | 39 | 4.730 | **0.030** |  | 38 | 33 | 0.030 | 0.863 |
| ≤58 | 15 |  | 10 | 5 |  |  |  | 7 | 8 |  |  |  | 12 | 3 |  |  |  | 9 | 6 |  |  |
| Tumor size |  |  |  |  |  |  |  |  |  |  |  |  |  |  |  |  |  |  |  |  |  |
| >4.8 | 52 |  | 37 | 15 | 0.367 | 0.545 |  | 19 | 33 | 0.042 | 0.837 |  | 25 | 27 | 0.238 | 0.626 |  | 26 | 26 | 0.722 | 0.395 |
| ≤4.8 | 34 |  | 27 | 7 |  |  |  | 14 | 20 |  |  |  | 19 | 15 |  |  |  | 21 | 13 |  |  |
| Pathological stage |  |  |  |  |  |  |  |  |  |  |  |  |  |  |  |  |  |  |  |  |  |
| I-II | 72 |  | 55 | 17 | 0.902 | 0.342 |  | 24 | 48 | 4.749 | **0.029** |  | 36 | 36 | 0.039 | 0.844 |  | 38 | 34 | 0.248 | 0.618 |
| III-IV | 14 |  | 9 | 5 |  |  |  | 9 | 5 |  |  |  | 8 | 6 |  |  |  | 9 | 5 |  |  |
| T stage |  |  |  |  |  |  |  |  |  |  |  |  |  |  |  |  |  |  |  |  |  |
| T1-T2 | 5 |  | 5 | 0 | 1.743 | 0.187 |  | 1 | 4 | 0.705 | 0.401 |  | 3 | 2 | 0.188 | 0.664 |  | 3 | 2 | 0.074 | 0.786 |
| T3-T4 | 80 |  | 59 | 21 |  |  |  | 31 | 49 |  |  |  | 40 | 40 |  |  |  | 43 | 37 |  |  |
| AJCC stage |  |  |  |  |  |  |  |  |  |  |  |  |  |  |  |  |  |  |  |  |  |
| I-II | 53 |  | 38 | 15 | 0.229 | 0.632 |  | 19 | 34 | 0.146 | 0.703 |  | 23 | 20 | 0.427 | 0.513 |  | 26 | 27 | 1.206 | 0.272 |
| III-IV | 33 |  | 26 | 7 |  |  |  | 14 | 19 |  |  |  | 21 | 12 |  |  |  | 14 | 19 |  |  |

Bold signifies P <0 .05.

**Supplementary Table 3 Correlations of CD8^+^、CXCL13^+^CD8^+^、CXCL13^+^PANCK^+^、CXCL13^+^IL21^+^CD4^+^ expression with clinicopathological parameters in colon cancer patients**
